# Nécroses cutanées multiples et extensives révélant un syndrome des anticorps anti phospholipids

**DOI:** 10.11604/pamj.2016.25.56.10572

**Published:** 2016-09-30

**Authors:** Mohamed El Amraoui, Hafsae Bounnyit, Youssef Zemmez, Ahmed Bouhamidi, Baderdine Hassam, Karima Senouci

**Affiliations:** 1 Service de Dermatologie Vénéréologie, CHU Ibn Sina, Rabat, Maroc

**Keywords:** Nécroses cutanées, syndrome des anticorps anti phospholipides, lupus érythémateux systémique, Skin necrosis, anti phospholipid antibody syndrome, lupus erythematosus

## Abstract

Le syndrome des anticorps anti phospholipides (SAPL) est un état de thrombophilie acquise, du à l'action des auto-anticorps dirigés contre les phospholipides et/ou leurs cofacteurs. Il peut être primitif ou associé à diverses maladies, dont le lupus érythémateux systémique (LES), dont il représente un facteur potentiel de gravité. Nous rapportant un cas de multiples nécroses cutanées chez une jeune fille ayant révélé un SAPL dans le cadre d'un LES. Les lésions dermatologiques au cours du SAPL sont fréquentes, polymorphes parfois inaugurales et peuvent constituer le seul élément clinique du syndrome. Cependant, les nécroses cutanées sont rares, leur traitement repose sur les anticoagulants et les soins locaux appropriés. L'évolution ultérieure imprévisible et le pronostic réservé justifient un suivi rigoureux au long cours et une étroite collaboration entre le dermatologue et l’interniste.

## Abstract (en)

Antiphospholipid antibody syndrome (APS) is an acquired thrombophilic state resulting from autoantibodies activity against phospholipides and/or their cofactors. It may be primary or associated with various diseases, including systemic lupus erythematosus (SLE), which represents a potential severity marker. We here report the case of a young girl with multiple cutaneous necrosis suffering from APS secondary to SLE. Skin lesions associated with APS are common, polymorphic, sometimes inaugural and may be the only clinical manifestation of the syndrome. However, skin necrosis are rare; their treatment is based on anticoagulation therapy and appropriate local care. Rigorous long term follow up associated with closer cooperation between the dermatologist and the internist is justified by unpredictable outcomes and poor prognosis.

## Introduction

Le syndrome des anticorps anti phospholipides (SAPL) est un état de thrombophilie acquise, du à l’action des auto-anticorps dirigés contres les phospholipides et/ou leurs cofacteurs. Il peut être primitif ou associé à diverses maladies, dont le lupus érythémateux systémique (LES), dont il représente un facteur potentiel de gravité. Nous rapportons un cas de nécroses cutanées multiples chez une jeune fille ayant révélé un SAPL dans le cadre d’un LES.

## Patient et observation

**Figure 1:**
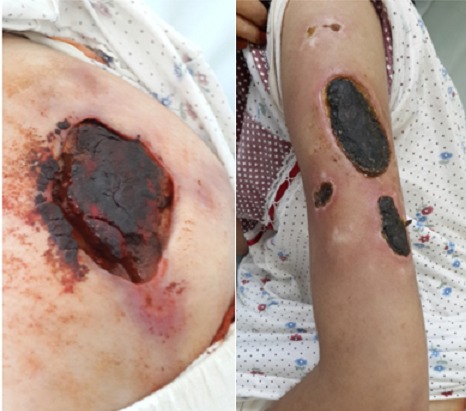
Nécroses cutanées au niveau de la fesse gauche et du bras gauche

**Figure 2:**
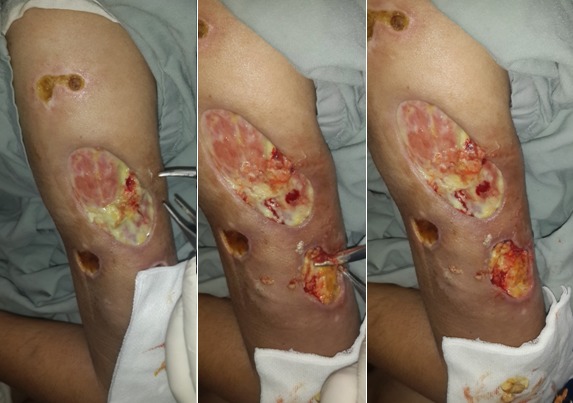
Exérèse chirurgicale des foyers de nécrose

**Figure 3:**
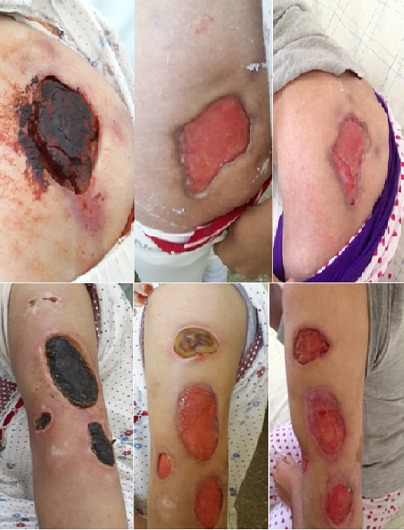
Evolution des nécroses après 15 jours et après 1 mois

Une patiente de 20 ans qui avait comme antécédents une épilepsie, un lupus érythémateux chronique et des érosions buccales récidivantes, a été admise au service de dermatologie pour prise en charge de multiples nécroses cutanées extensives siégeant sur la fesse gauche et les deux bras et évoluant depuis un mois et demi dans un contexte fébrile avec une altération de l’état général (Figure 1). L’examen clinique objectivait également une arthrite du poignet droit et un gros membre inferieur gauche et une livedo racemosa. Le bilan paraclinique montrait un syndrome inflammatoire biologique important : une anémie normo chrome normocytaire à 8,5 g/dl, une vitesse de sédimentation à 130 mm/1ère heure, une protéine C réactive à 115 mg/l. Le bilan immunologique révélait des anticorps anti nucléaires positifs à 1280 UI/ml, des anticorps anti DNA natifs positifs et des anticorps anti coagulants circulants de type lupiques positifs. Le doppler veineux des membres inferieurs mettait en évidence une thrombophlébite fémoro-poplitée gauche et la biopsie cutané montrait une nécrose kératinocytaire étendue et une vascularite lympho-plasmocytaire. Le diagnostic du SAPL secondaire à une maladie lupique a été retenu et la patiente a été mise sous corticothérapie générale, anticoagulants, antibiothérapie et soins locaux avec détersion des foyers de nécrose (Figure 2). L’évolution était favorable avec un recul de 18 mois (Figure 3).

## Discussion

Le syndrome des anticorps anti phospholipides (SAPL) est un état de thrombophilie acquise, du à l´action des auto-anticorps dirigés contre les phospholipides et/ou leurs cofacteurs. Il peut être primitif ou associé à diverses maladies, dont le lupus érythémateux systémique, dont il représente un facteur potentiel de gravité. Son diagnostic repose sur les critères de Sapporo établis en 1999 et révisés en 2006 [1]. Les lésions dermatologiques au cours du SAPL sont fréquentes, polymorphes, parfois inaugurales et peuvent constituer le seul élément clinique du syndrome [2, 3]. Cependant, les nécroses cutanées superficielles extensives restent extrêmement rares, rapportées seulement dans 2% environ des cas de SAPL [3, 4]. Leur début est volontiers brutal avec un purpura nécrotique, laissant rapidement place à une plaque escarrotique noirâtre bordée d’un liseré purpurique témoignant de leur évolutivité. Elles siègent sur les membres, le visage (joues, nez, oreilles) ou les fesses comme chez notre patiente. Leur traitement est préventif et curatif; l’indication d’une anti coagulation efficace est incontournable, éventuellement en association avec des corticoïdes, immunosuppresseurs, échanges plasmatiques ou biothérapies, de même, la détersion des tissus nécrosés est primordiale pour la prévention des surinfections, rappelons enfin l’importance de la prise en charge d’éventuels facteurs de risque thrombotiques associés, présents chez environ 50% des patients ayant un SAPL [5].

## Conclusion

Les nécroses cutanées au cours du SAPL sont extrêmement rares, leur traitement repose sur les anticoagulants et les soins locaux appropriés. L’évolution ultérieure imprévisible et le pronostic réservé justifient un suivi rigoureux au long cours et une étroite collaboration entre le dermatologue et l'interniste.
